# Genome-wide transcriptome profiling provides overwintering mechanism of *Agropyron mongolicum*

**DOI:** 10.1186/s12870-017-1086-3

**Published:** 2017-08-10

**Authors:** Jiancai Du, Xiaoquan Li, Tingting Li, Dongyang Yu, Bing Han

**Affiliations:** 1 Institute of Grassland Research of Chinese Academy of Agricultural Sciences, Hohhot, China; 2 College of Life Sciences Inner Mongolia Agricultural University, No. 306 Hohhot Zhao Wuda Road, Hohhot, China

**Keywords:** *Agropyron mongolicum*, Genome-wide transcriptome, Overwintering mechanisms, Perennial gramineous plants

## Abstract

**Background:**

The mechanism of winter survival for perennials involves multiple levels of gene regulation, especially cold resistance. *Agropyron mongolicum* is one important perennial grass species, but there is little information regarding its overwintering mechanism. We performed a comprehensive transcriptomics study to evaluate global gene expression profiles regarding the winter survival of *Agropyron mongolicum*. A genome-wide gene expression analysis involving four different periods was identified. Twenty-eight coexpression modules with distinct patterns were performed for transcriptome profiling. Furthermore, differentially expressed genes (DEGs) and their functional characterization were defined using a genome database such as NT, NR, COG, and KEGG.

**Result:**

A total of 79.6% of the unigenes were characterized to be involved in 136 metabolic pathways. In addition, the expression level of ABA receptor genes, regulation of transcription factors, and a coexpression network analysis were conducted using transcriptome data. We found that ABA receptors regulated downstream gene expression by activating bZIP and NAC transcription factors to improve cold resistance and winter survival.

**Conclusion:**

This study provides comprehensive transcriptome data for the characterization of overwintering-related gene expression profiles in *A. mongolicum*. Genomics resources can help provide a better understanding of the overwintering mechanism for perennial gramineae species. By analyzing genome-wide expression patterns for the four key stages of tiller bud development, the functional characteristics of the DEGs were identified that participated in various metabolic pathways and have been shown to be strongly associated with cold tolerance. These results can be further exploited to determine the mechanism of overwintering in perennial gramineae species.

**Electronic supplementary material:**

The online version of this article (doi:10.1186/s12870-017-1086-3) contains supplementary material, which is available to authorized users.

## Background

Damage secondary to cold temperatures is one of the most severe environmental stresses that limits plant growth and yield. Low temperature damage is mainly caused by changes in the phospholipid bilayer membrane, especially conformational space and physical state. The formation of ice crystals leads to not only excessive protoplasmic dehydration and protein denaturation, but also mechanical damage to plant cells.

Previous research regarding the mechanisms of plant cold hardiness demonstrated that plants can improve their cold resistance mainly in the following four ways: (1) increasing the variety of proteins induced by low temperature; (2) upregulating the expression of multiple cold resistance gene without control through the DREB/CBF transcription factor; (3) regulation of low temperature–induced genes by Ca^2+^ and ABA; and (4) increasing the expression of unsaturated fatty acid enzymes. Proteins induced by low temperatures are functional proteins that improve plant cold hardiness and protect cells from the cold. These include antifreeze proteins (AFP), DNA-binding proteins, and mRNA-binding proteins such as bZip proteins and other related enzymes [1]. Dozens of cold-induced genes have been isolated from arabidopsis, rice, and other plants [2]. Since Stockinger [3] first cloned AtCBF1 from arabidopsis cDNA libraries, cloning of DREB/CBF transcription factor genes has been performed to better understand its functions. At present, DREB/CBF has been cloned from *Oryza sativa* [4], *Glycine max* [5], *Brassica napus* [6], and *Zea mays* [7]. The over-expression of DREB/CBF can enhance plant cold hardiness [8–10]. In the early 1980s, ABA received attention for its relevance with plant cold hardiness. *Arabidopsis* los5 and los6 [11] mutants with a decreased ABA level are more sensitive to low temperatures than wild types. At present, the role of ABA in plant cold resistance has been confirmed in many kinds of plants [12–19]. Research on the effect of membrane phosphatidylglycerol fatty acids on plasma composition, as well as on the relationship with cold hardiness, in 74 plant species showed that plasma membrane unsaturated fatty acid has a great influence on cold hardiness. Higher unsaturated fatty acid content increases plant cold hardiness [20]. In addition, genetic manipulation of fatty acid desaturation changes the sensitivity of plants with low temperature stress [21]. Cold hardiness is indispensable for the winter survival of perennial plants. Therefore, the process for plants overwintering involves multiple genes. This multi-level network regulation is complex.

*Agropyron mongolicum*, a perennial grass, is distributed in Eurasian steppe, mainly in Russia, Mongolia, and northern China [22]. *A. mongolicum* has a high nutritional value with a protein content of 18.64%. Its stem leaves are soft with good palatability for animal husbandry. It also exhibits high ecological value because of its drought resistance, cold resistance, and suitability for dry sandy areas. *A. mongolicum* belongs to the wild relatives of *Triticum*. Its cold resistance, drought resistance, salt tolerance, resistance to diseases and pests, and excellent resistance genes provide a genetic resource for genetic modification of food crops (such as wheat and barley). Many perennial plants can sexually reproduce and clone growth at the same time [23]. These plants are called clonal plants [24]. The seed development rate of *A. mongolicum* is low, and studies have shown that the fruit set rate is only 43.45% under suitable conditions. Therefore, it is a typical clonal plant that has new plant generation mainly from the mother plant through an underground horizontal rhizome. This type of root system is advantageous for *A. mongolicum*, as it is able to fully use soil nutrients and moisture from the space, providing structural basis for plant growth and development.

**Fig. 1:**
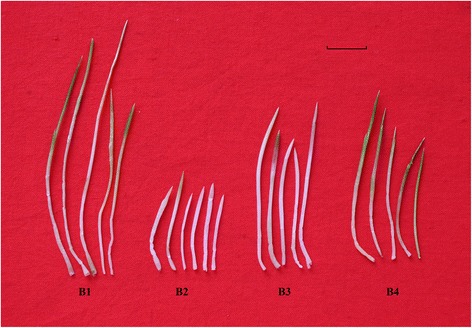
Morphological characteristics of tiller buds during four stages. B1, B2, B3, and B4 represent normal growth period, before winter, winter, and after turning green, respectively. The scale is 1 cm

*A. mongolicum* from northern China was used in the present study. Comprehensive transcriptomics was conducted to evaluate global gene expression profiles during four stages including normal growth period (October 5, 2014), before winter (November 1, 2014), during winter (January 31, 2015), and after reviving (March 16, 2015). For our sample collection, the studied time points included normal growth, lowest temperature, and return to green, as shown in Fig. 1. We found a large number of different expression genes (DEGs), and our weighted gene co-expression network analysis demonstrated 28 modules. This study helps augment our knowledge of wintering mechanisms.

## Results

### Morphological characteristics of tiller bud development

**Fig. 2:**
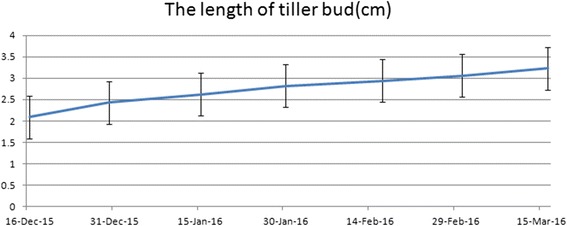
Tiller bud length in winter. Samples (*n* = 100) were obtained every 15 days

To obtain the genetic mechanism for overwintering in *A. mongolicum*, a comprehensive transcriptome analysis was performed using a next-generation sequencing platform. First, a morphological analysis was performed on tiller buds during their development process. The results are presented in Fig. 1. We collected bud samples from October 2014 to March 2015 and examined the length of tiller buds. We found that tiller bud length still increased between the B2 and B4 periods despite entering the winter season (Fig. 2). Thus, we decided to determine the mechanism of *A. mongolicum* wintering using a transcriptome sequencing method.

The development of tiller buds was in line with bud size (Fig. 1), and we identified four stages of tiller bud development, including normal growth period (on October 5, 2014), before winter (on November 1, 2014), during winter (on January 31, 2015), and after reviving (on March 16, 2015). First-stage tiller buds (normal growth period) were green and more than 4–6 cm; second-stage buds (before winter) were white and buried underground with bud sizes of approximately 2 cm; third-stage buds (during winter) were 1–2 mm above the ground with bud sizes of approximately 3–4 cm; and final-stage buds (after reviving) were 1–2 cm above the ground and turned green with bud sizes of approximately 4–5 cm.

### De novo assembly and sequence annotation

**Fig. 3:**
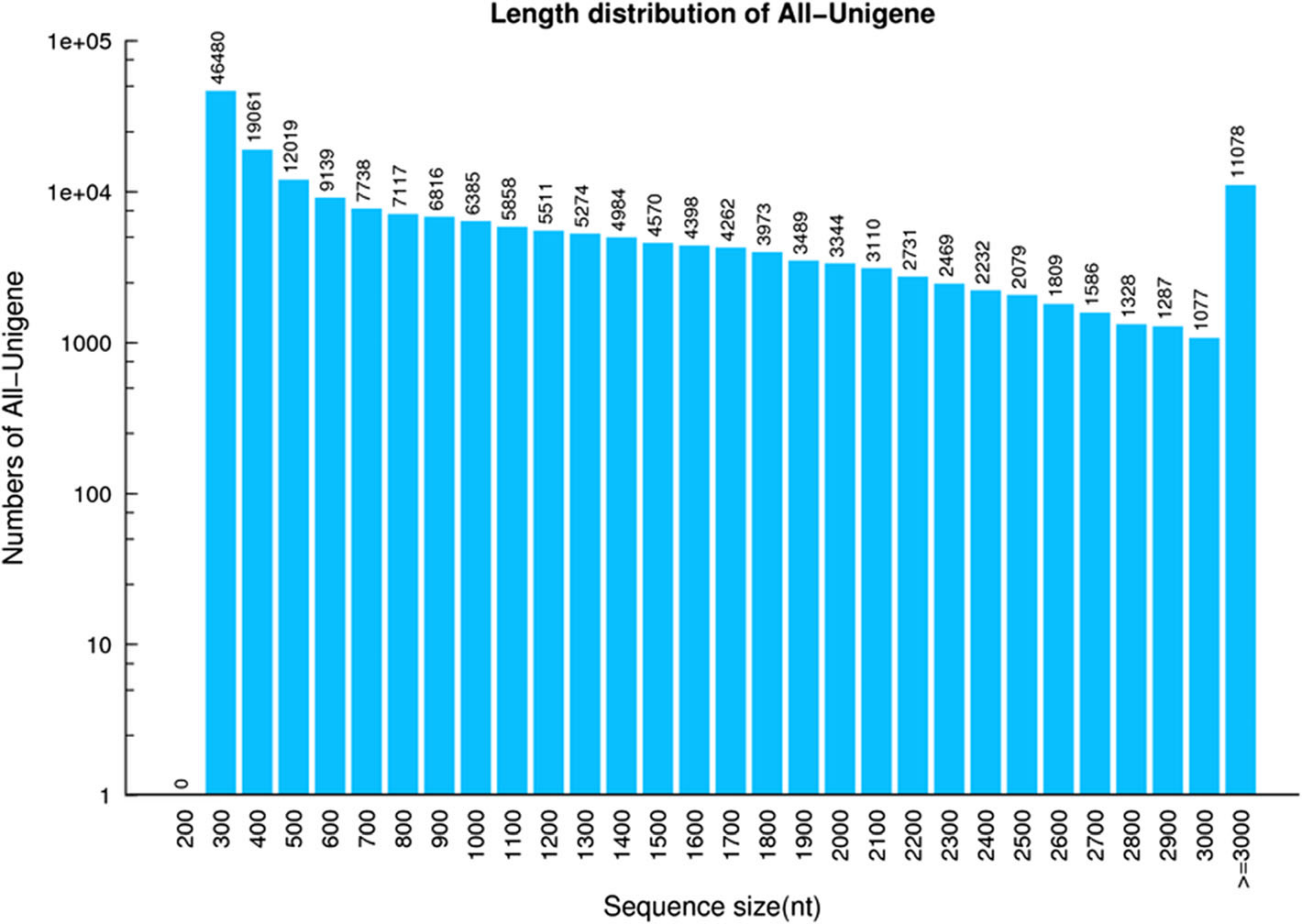
Distribution of unigenes. X axis represents length of unigenes. Y axis represents the number of unigenes

The total RNA in each sample was extracted and tested using an Agilent 2100 bioanalyzer. The total RNA with an RIN value >8 was used to build a cDNA library, which was then sequenced using an Illumina HiSeq 4000 system; about 66.33 Gb bases were generated from the four stages of *A. mongolicum* (B3 stage had two repeats; B1, B2, and B4 stages had 3 repeats). After filtering low-quality, adaptor-polluted, and high content from unknown base (N) reads, clean reads were generated. After assembling all samples, 191,204 unigenes were obtained, with a total length, an average length, N50, and GC content of 208,509,575 bp; 1090 bp; 1811 bp; and 50.36%, respectively (Table 1). The size distribution for these unigenes is presented in Fig. 3. The assembly produced a substantial number of large unigenes; the 82,834 unigenes were >1000 bp in length.

**Table 1:** Quality metrics of Unigenes

Sample	Total Number	Total Length	Mean Length	N50	N70	N90	GC(%)
B1-1	58216	51926661	891	1465	907	353	51.78
B1-2	61352	54707935	891	1466	900	354	52.05
B1-3	57648	52116217	904	1467	921	362	51.96
B2-1	57781	48810094	844	1400	852	327	50.6
B2-2	54719	45107200	824	1366	822	319	51.27
B2-3	57956	49662741	856	1424	868	333	50.43
B3-1	67819	48701817	718	1143	662	283	51.73
B3-2	74266	54664602	736	1209	690	284	50.47
B4-1	58979	50849997	862	1445	878	334	51.74
B4-2	60744	53423598	879	1493	911	334	50.47
B4-3	61409	53566582	872	1503	904	329	50.11
All-Unigene	191204	208509575	1090	1811	1197	456	50.36

N50: a weighted median statistic that 50% of the TotalLength is contained in transcripts great than or equal to this value. GC (%): the percentage of G and C bases in all transcripts.

**Fig. 4:**
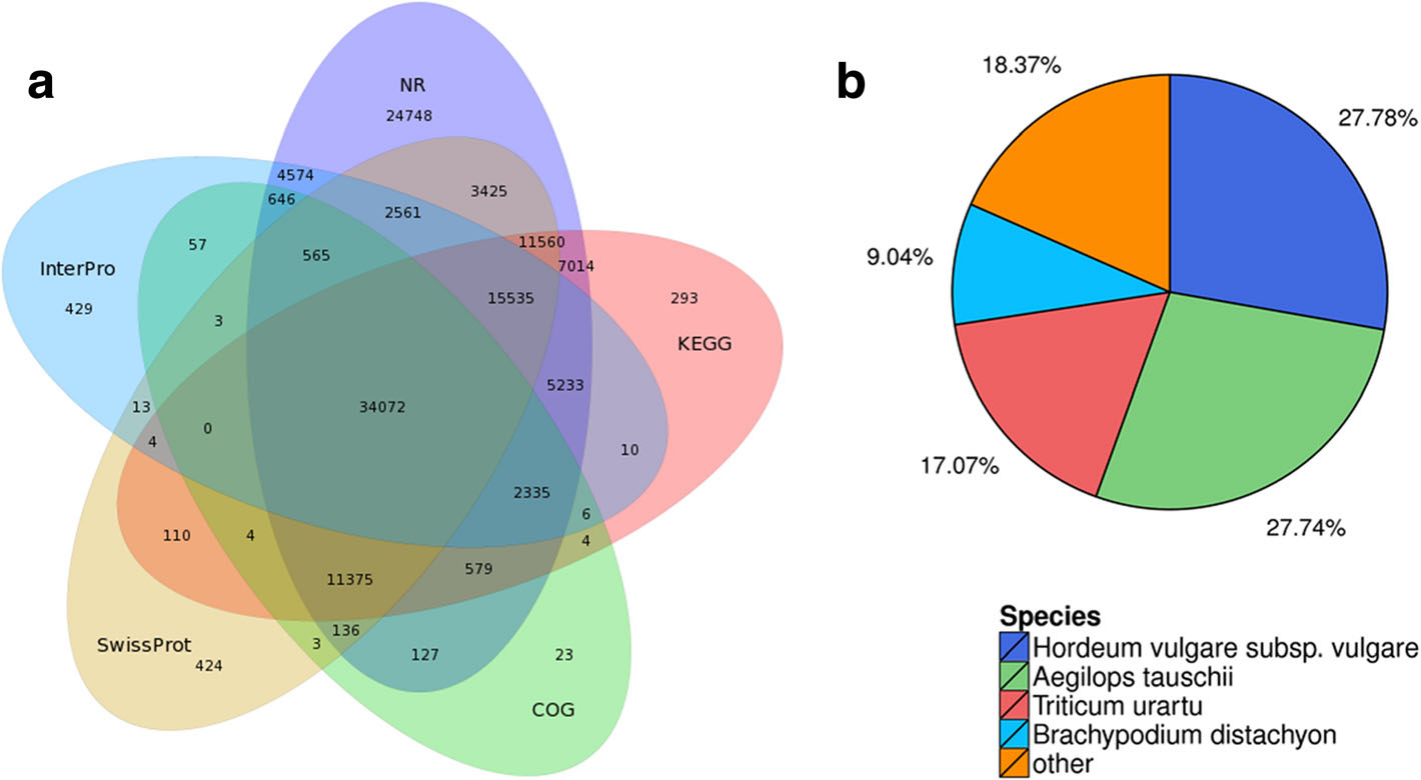
**a** Venn diagram of the number of unigenes annotated by BLASTx with a cut-off E-value 1e^−05^ against protein databases. The numbers in the circles indicate the number of unigenes annotated by single or multiple databases; **b** Distribution of annotated species. Numbers represent the percentage of unigenes annotated to species accounted for by all unigenes

The unigenes were annotated using seven functional databases. In total, 152,241 (79.62%) unigenes were annotated as follows: 124,485 (NR: 65.11%), 138,132 (NT: 72.24%), 79,790 (Swissprot: 41.73%), 49,935 (COG: 26.12%), 88,134 (KEGG: 46.09%), 38,285 (GO: 20.02%), and 66,043 (Interpro: 34.54%) (Fig. 4a). Using NR annotation, the distribution for the annotated species is shown in Fig. 4b, and 27.78%, 27.74%, 18.37%, and 9.04% of all unigenes were annotated to *Hordeum vulgare*, *Aegilops tauschii*, *Triticum urartu*, and *Brachypodium distachyon*, respectively.

**Fig. 5:**
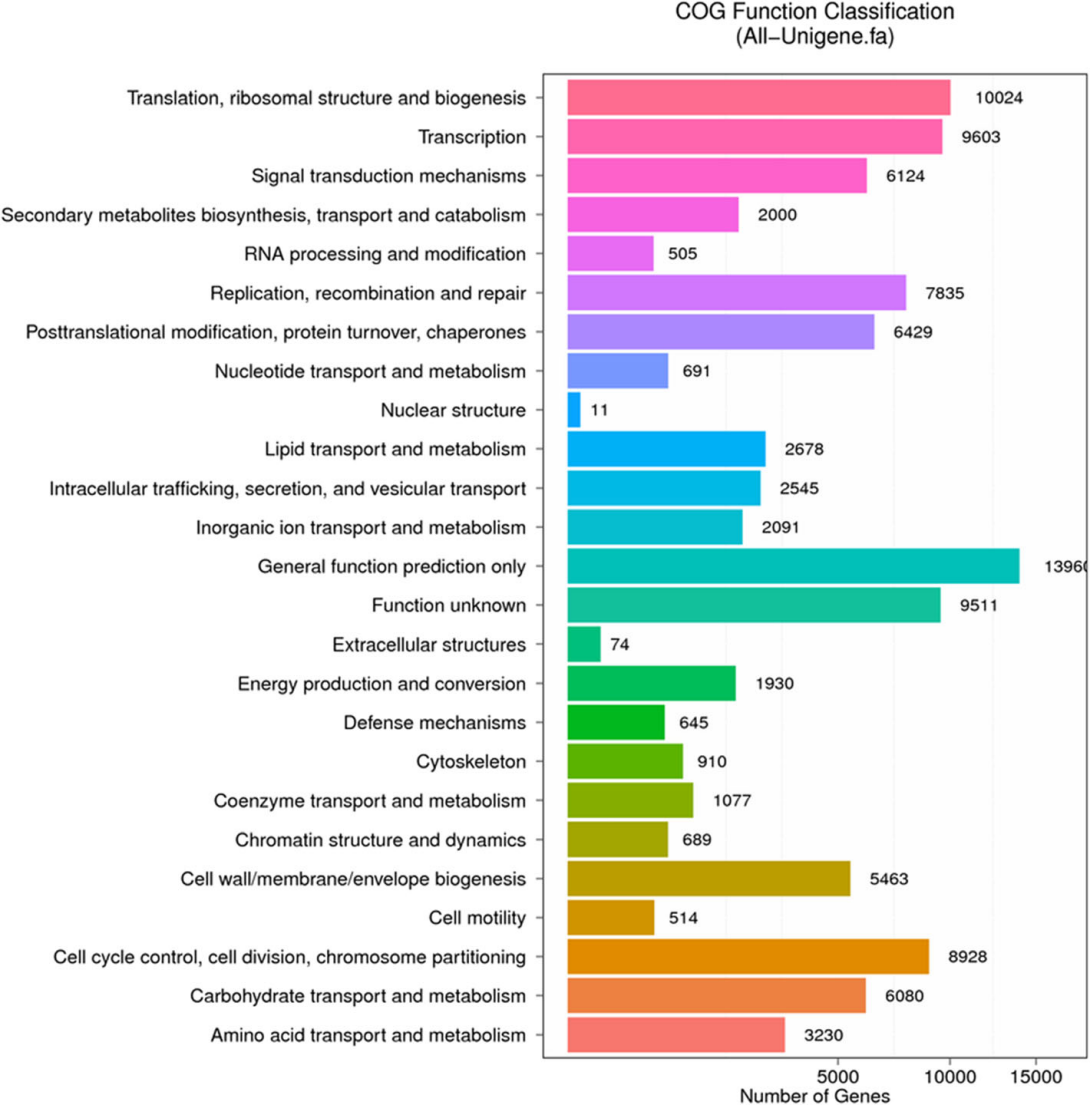
Functional distribution of COG annotation. X axis represents the number of unigenes. Y axis represents COG functional category. A total of 191,204 unigenes were classified into 25 functional categories according to their predicted gene products using the COG database

Among the 152,241 unigenes, approximately 26.12% could be annotated using COG based on sequence homology, and of them, 49,935 Unigenes were classified into 25 functional classifications (Fig. 5). The dominant term was “general function prediction only,” and 13,960 unigenes (28%) matched it. “Translation,” “transcription,” and “cell-cycle control, cell division, chromosome partitioning” also shared a high percentage of genes among the categories; 11 and 74 unigenes matched the terms “nuclear structure” and “extracellular structures,” respectively. Meanwhile, 6124 unigenes were annotated as the “signal transduction mechanisms” category, and 2545 unigenes were annotated as the “intracellular trafficking, secretion, and vesicular transport” category; both of them might play an important role in signal transduction.

### GO and pathway analysis

The functions for the predicted *A. mongolicum* unigenes were classified using GO assignment. A total of 38,285 unigenes were divided into 57 functional GO items (Additional file 1: Figure S1). Among these, 23, 17, and 17 items were involved in biological processes, cellular components, and molecular functions, respectively. Metabolic and cellular processes were the dominant biological process items. A high percentage of genes was associated with catalytic activity and binding with respect to the molecular function category. Most cellular component assignments were related to cell components and membranes (Additional file 1: Figure S1).

A pathway analysis was primarily based on the KEGG database and was used to classify functional annotations for all annotated sequences. In total, 88,134 sequences were classified to 136 pathways. The metabolic pathways were dominant (50,512 unigenes, 57.3%), with most unigenes involved in carbohydrate metabolism (6859 unigenes). “Plant hormone signal transduction” and “phosphatidylinositol signaling system” also shared numerous genes among the categories, with 2169 and 606 unigenes, respectively. A total of 5729 unigenes were involved in the biosynthesis of secondary metabolites, including phenylpropanoid biosynthesis, flavonoid biosynthesis, stilbenoid, diarylheptanoid and gingerol biosynthesis, isoflavonoid biosynthesis, etc. (Additional file 2: Figure S2. Analysis of these pathways provided a valuable resource for investigating specific processes, functions, and pathways regarding the wintering of *A. mongolicum*.

Hormones have been proven to play an important role in the process of plant development during the winter. A KEGG pathway analysis demonstrated that hormonal regulations contained 2169 unigenes (Additional file 3: Figure S3). The pathway analysis results will be useful for additional study on how hormone regulation occurs in *A. mongolicum* through a cold winter.

### Identification of DEGs and transcription factors

**Fig. 6:**
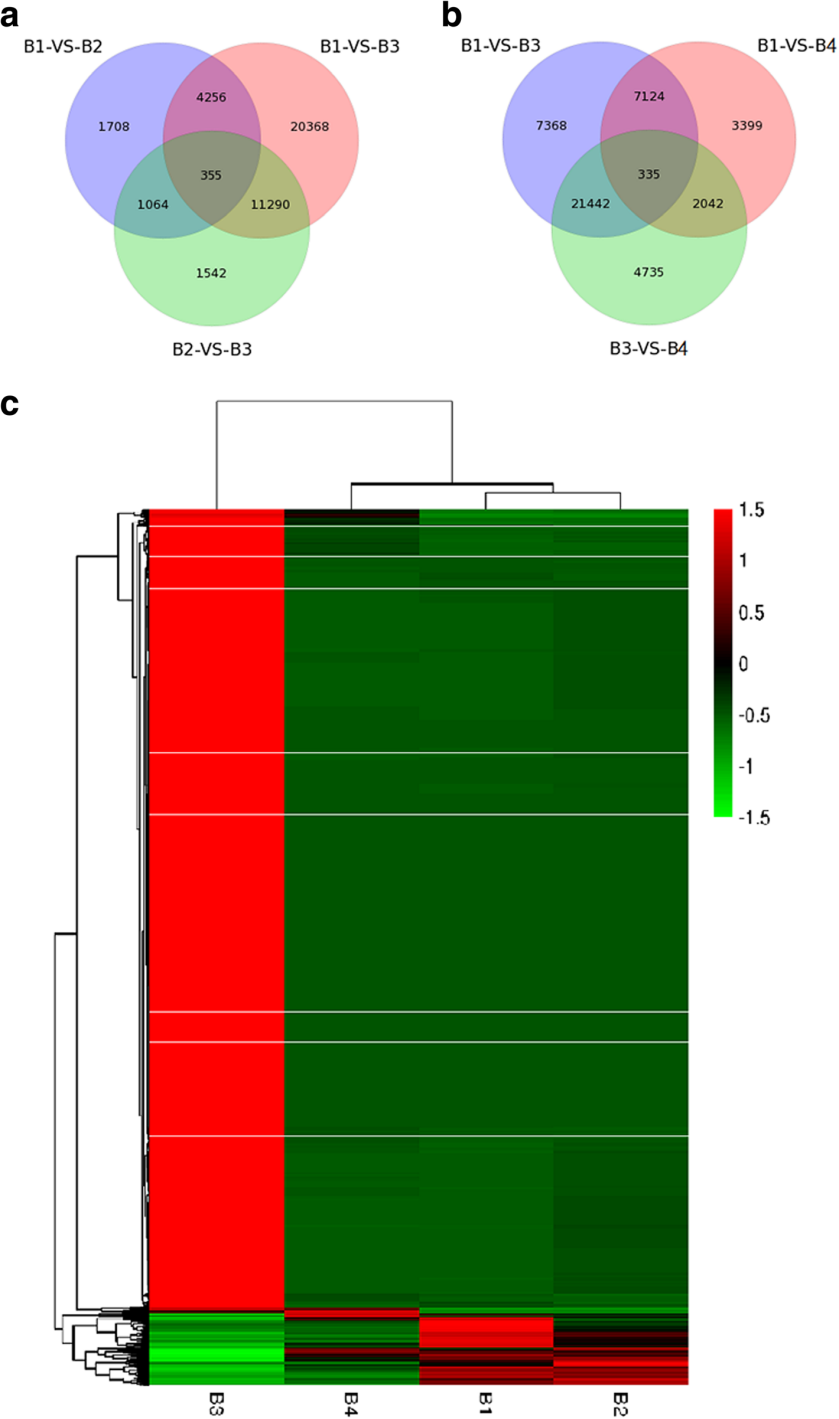
Analysis of DEGs between four developmental stages. **a** Venn diagram of the distribution of DEGs at B1, B2, and B3. **b** Venn diagram of the distribution of DEGs at B1, B3, and B5. **c** A hierarchical cluster graph of DEGs found between B1–B3 and B2–B3, but not B1–B2. Each column represents an experimental sample (e.g., B1, B2, B3, and B4), and each row represents a gene. Expression differences are shown in different colors. Red indicates high expression, and green indicates low expression

**Fig. 7:**
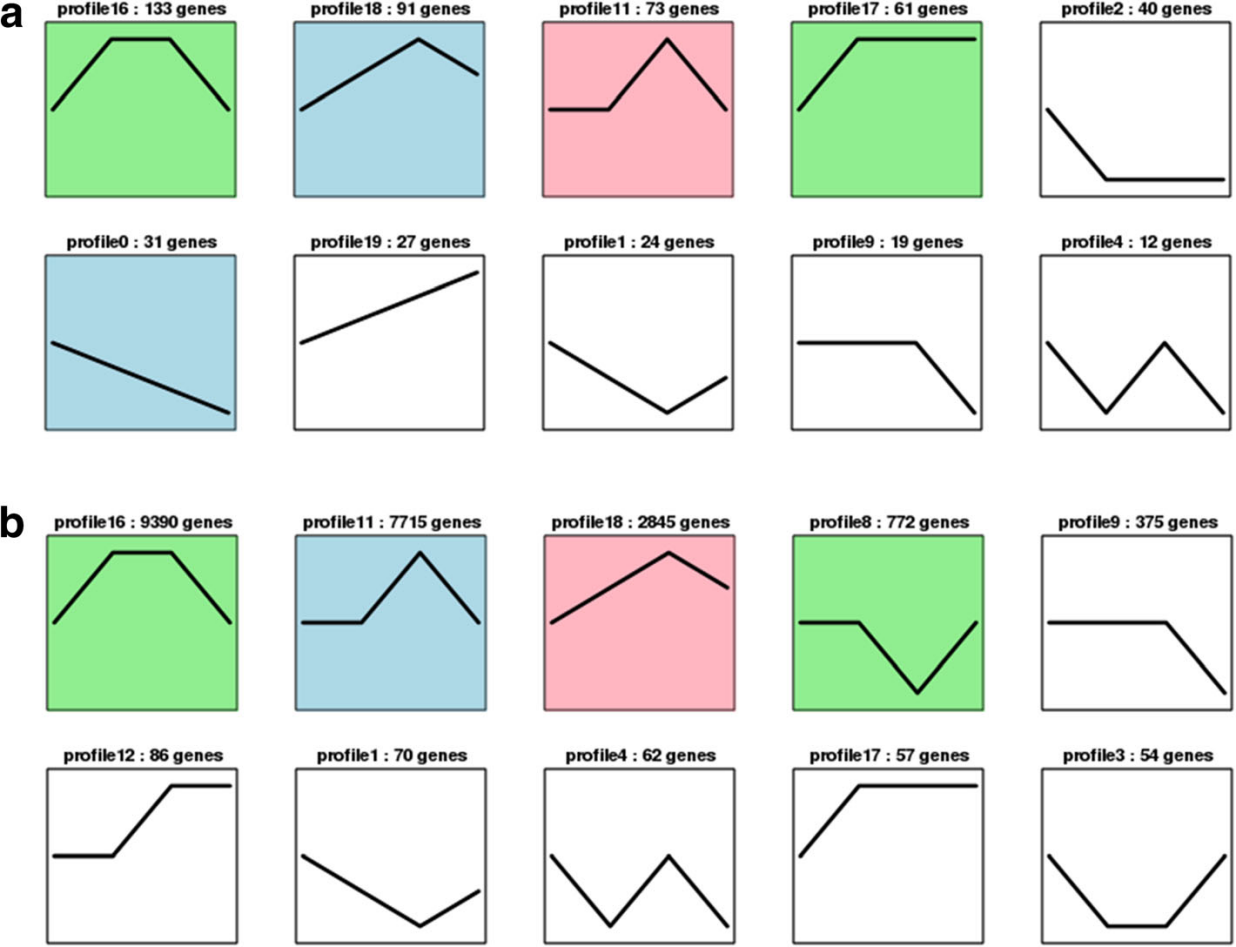
Trend analysis of gene expression. **a** Trend analysis of TFs (transcription factor). **b** Trend analysis of DEGs found between B1–B3 and B3–B5, but not B1–B5. Colored block trend: significant enrichment trend. Without color trend: the enrichment of significant trends

To investigate expression differences between distinct developmental stages, the DEGs were identified by applying a cutoff *p*-value of <0.05 (FDR Bonferroni corrected). The DEG distribution is presented in Fig. 6, and there were 7383, 14,251, and 36,269 DEGs in B1–B2, B2–B3, and B1–B3, respectively (Fig. 6a). The number of DEGs in B2–B3 and B1–B3 was larger than in B1–B2 indicating the involvement of complex developmental events during stage B3. The DEGs between B1–B3 and B2–B3 overlapped and contained 11,290 unigenes (Fig. 6a), suggesting that it was specifically involved in the developmental processes at stage B3. To characterize this portion in detail, this group of genes was further clustered. Two major clusters of expression patterns were demonstrated; the first one had the highest apparent expression at stage B3, and the second had the lowest expression levels at stage B3 (Fig. 6c). This phenomenon was related to the sampling period. With these unigenes, we performed a KEGG pathway classification and found that the terms “RNA transport,” “metabolic pathways,” “biosynthesis of secondary metabolites” had stronger enrichment than the others (Additional file 4: Figure S4), indicating that these biological processes play an important role. The other Venn diagram shows that there were 36,269, 28,554, and 12,900 DEGs in B1–B3, B3–B4, and B1–B4, respectively (Fig. 6b). The DEGs between B1–B3 and B3–B4 overlapped and contained 21,442 unigenes. Interestingly, changes in gene expression quantity demonstrated an opposite trend (Fig. 7b). There were 19,950 gene expressions that first rose after falling, and 772 genes demonstrated the opposite trend. In addition, two cases reached the enrichment condition (*p* < 0.05). With these unigenes, we performed a KEGG pathway classification and found that the terms for “mRNA surveillance pathway” and “RNA transport” were enriched (Additional file 5: Figure S5). These results indicated that these biological processes play an important role in winter survival, as compared with the normal growth condition.

Transcription factors (TFs) have been implicated in a variety of developmental and physiological roles. Moreover, TFs have also been isolated and characterized for several secondary plant metabolic pathways. In the present study, a total of 3537 putative transcripts encoding TFs were identified, and 652 gene expressions changed. With additional analysis of these genes, we performed a trend analysis with STEM [25] and found that 297 TF expressions first rose after falling; there were 61 TF expressions that first rose after holding the line, and 31 genes persistently declined (Fig. 7a). These transcription factors may exist in one biological process. They all belonged to already known TF families; the most abundant was the MYB family, including 84 unigenes. Meantime, 54 TFs belonged to the bHLH family; 51 belonged to the AP2/ERF family; 22 belonged to the WRKY family; and 42 belonged to the NAC family. All these TFs have been identified as regulators in the biosynthesis of secondary metabolites in other plants [26, 27].

ABA plays an important role in cold resistance in plants. In the annotation of plant hormone signaling pathways, 250 genes were annotated to the ABA signaling pathways. Among them, five genes were annotated to ABA receptor gene differences, including CL2537.Contig2_All, CL4533.Contig2_All, CL4558.Contig2_All, CL10173.Contig1_All, and CL16949.Contig3_All. These gene expressions first rose after falling, indicating that ABA receptor expression in *A. mongolicum* increased during the winter, opening the ABA signaling pathway and regulating the expression of downstream genes to improve cold resistance ability.

### DEGs related to ABA signaling pathways and qRT-PCR validation

To validate changes in gene expression patterns, 17 genes associated with hormone signal transduction, including AUXIAA (CL11192.Contig2_All, CL13005.Contig5_All, CL13845.Contig1_All, CL5043.Contig13_All, CL5043.Contig6_All), ABF (CL10642.Contig3_All, CL1142.Contig2_All, CL13456.Contig4_All), AUX1 (CL1176.Contig2_All), MYC2 (CL1179.Contig3_All), DELLA (CL19671.Contig3_All), TIR1 (CL3327.Contig3_All, Unigene41160_All), CTR1 (CL793.Contig7_All, Unigene32807_All), and TCH4 (Unigene15065_All, Unigene7745_All) were randomly selected for an examination of gene expression using qRT-PCR. There was a strong positive correlation (*r* = 0.906) between RNA-seq and the PCR data.

### Gene coexpression network analysis

**Fig. 8:**
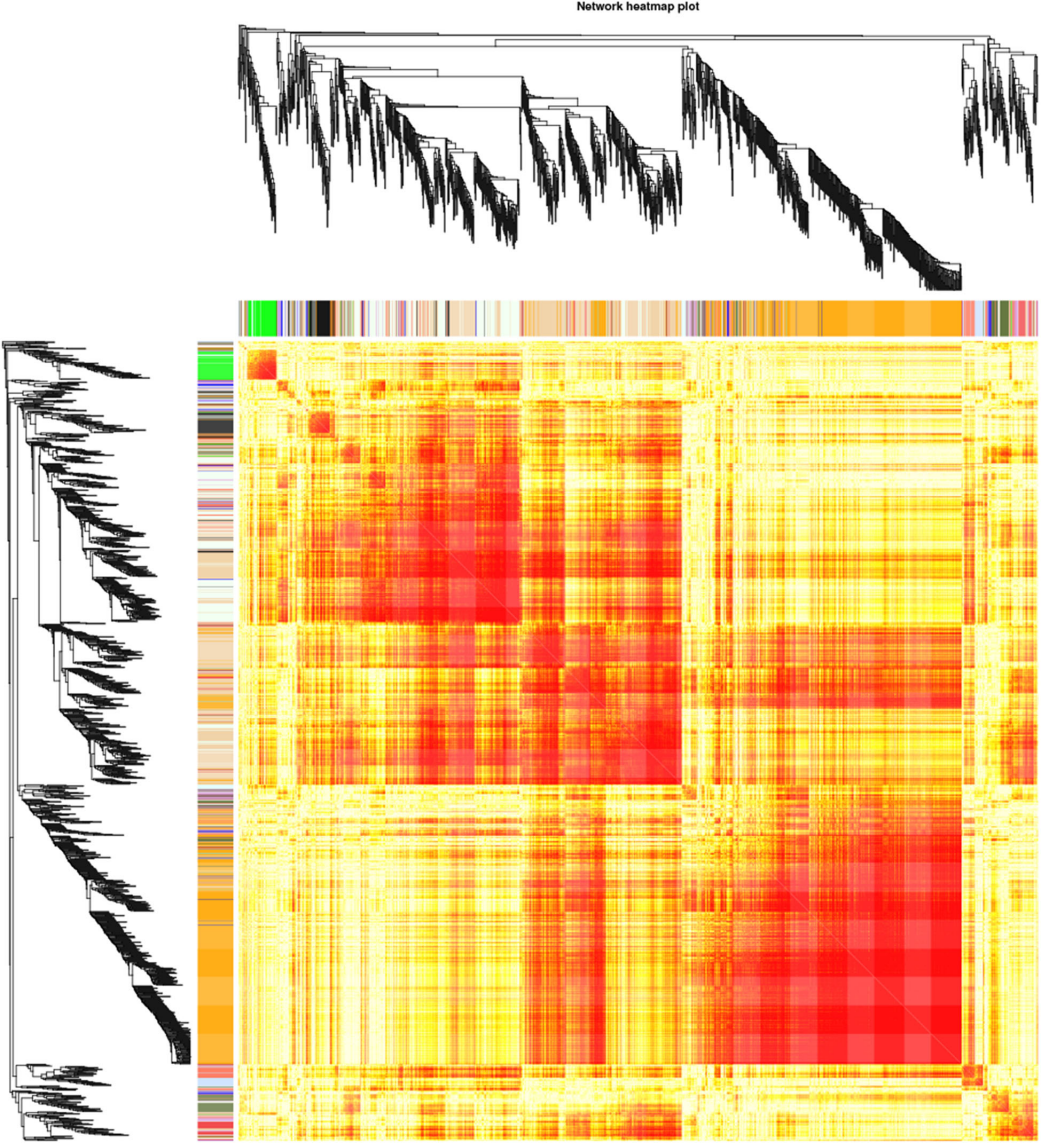
Heat map of correlation analysis among 28 modules. A different color represents different modules. Little correlation exists between any two modules, indicating that the module analysis result was reliable

Weighted gene coexpression network analysis (WGCNA) with scale-free topology can to use to study a biological problem systematically. This study identified 28 modules (according to the number of genes from lower to higher ordinal number, M1 to M28) containing 37,256 unigenes. The maximum number of genes was 11,102; 9 was the smallest; and the average for each module was 1330 genes. Genetic correlation between modules was smaller (Fig. 8), indicating that the module analysis result was reliable. These modules are likely to be associated with certain biological processes.

Module M20 had 787 genes; in addition, 313 and 384 unigenes were annotated in GO database and KEGG database, respectively. As for GO annotation (Additional file 6: Figure S6), the terms “lipid transport” and “lipid localization” indicated enrichment and that changes in fatty acid composition and content occur in *A. mongolicum* cells during winter. As for KEGG annotation (Additional file 7: Figure S7), the term “biosynthesis of other secondary metabolites” had the most enrichment. In addition, “unsaturated fatty acid metabolism” and “plant hormone signal transduction” also had a great proportion. This suggested that hormones such as ABA could improve the cold resistance of plants by adjusting secondary metabolites and unsaturated fatty acid metabolism.

## Discussion

**Fig. 9:**
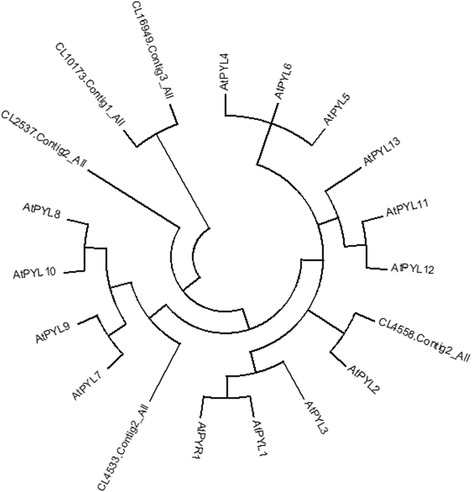
Phylogenetic analysis of ABA receptor from different plant species. Bootstrap values for 1000 replicates were used to assess the robustness of the trees. The genes are as follows: AtPYL1(Gene ID: 834,722), AtPYL2(Gene ID: 817,145), AtPYL3(Gene ID: 843,631), AtPYL4(Gene ID: 818,411), AtPYL5(Gene ID: 830,427), AtPYL6(Gene ID: 818,626), AtPYL7(Gene ID: 827,923), AtPYL8(Gene ID: 835,397), AtPYL9(Gene ID: 838,452), AtPYL10(Gene ID: 828,905), AtPYL11(Gene ID: 834,626), AtPYL12(Gene ID: 834,627), AtPYL13(Gene ID: 827,596), and AtPYR1 (Gene ID: 827,510)

Abiotic or biotic stresses simultaneously occur and severely affect the growth of plants in field environments [28]. Plant hormones are key regulators of tailored responses to different abiotic stresses in plants, such as drought and cold [29]. Using a yeast two-hybrid method, RCAR1 was found to mediate ABA-dependent inactivation of AB1 or AB12 in vitro (Ma et al., 2009) [30]. As ABA receptors, PYR/PYLs are involved in a negative regulatory pathway to control ABA signaling [31]. *A. mongolicum* is a perennial grass species that has a more complex regulatory mechanism for ABA signaling, as compared with other plants. In this study, a total of 250 genes were annotated to the ABA signaling pathways, and among them, five genes encoding the ABA receptors PYR/PYL were identified. Moreover, the PYL family belongs to a highly conserved protein family. Our results from an evolution analysis also provided evidence to support this [32] (Fig. 9).

Plant transcription factor families play crucial roles in multiple biological processes [33]. Basic leucine zipper transcription factors (bZIP) have been described in *Arabidopsis* based on the presence of conserved domains and gene structure [34]. Plant-specific NAC transcription factors play diverse roles in plant development and stress responses [35, 36]. bZIP and NAC transcription factor genes can be induced by ABA, drought, and cold stress. For example, expression of the OsbZIP52 gene was strongly induced by a low temperature (4 °C) [37]. Hu et al. (2016) reported that MebZIP genes are involved in the strong resistance of cassava to drought [38]. Similarly, previous studies have indicated that an NAC (NAM, ATAF1/2, and CUC2) transcription factor (TF) gene can be induced by cold tolerance in various plants, such as *Arabidopsis* [39], rice [40], wheat [41], and banana [42]. Moreover, previous studies have confirmed that ABA can activate bZIP transcription factors [43]. In our study, nine bZIP transcription factor genes and 42 NAC TFs were identified, and changes in gene expression demonstrated significant differences during the four stages that might play an important role in the induction of cold resistance in *Agropyron mongolicum*.

## Conclusions

We performed a comprehensive transcriptomics study to evaluate global gene expression profiles regarding the winter survival of *Agropyron mongolicum*. A genome-wide gene expression analysis involving four different periods was identified. A total of 79.6% of the unigenes were characterized to be involved in 136 metabolic pathways. In addition, the expression level of ABA receptor genes, regulation of transcription factors, and a coexpression network analysis were conducted using transcriptome data. We found that ABA receptors regulated downstream gene expression by activating bZIP and NAC transcription factors to improve cold resistance and winter survival. These results can be further exploited to determine the mechanism of overwintering in perennial gramineae species.

## Methods

### Plant materials and RNA extraction

Seeds of *Agropyron mongolicum* (saved number: 05346) were collected from Bayannur of Inner Mongolia, which was in good accordance with local legislation, no specific permission was needed. The formal identification of seeds was performed by National mid-term forage germplasm repository. *A. mongolicum* was grown at the Inner Mongolia Agricultural University of China. Tiller buds were collected at different time points (October 5, 2014; November 1, 2014; January 31, 2015; and March 16, 2015) and then frozen immediately in liquid nitrogen and stored at −80 °C. Total RNA for each sample was extracted using a Column Plant RNAout2.0 kit (TIANDZ, China) according to the manufacturer’s instructions. Column DNA Erasol (TIANDZ, China) was used to avoid DNA contamination. The quality and quantity of total RNA were determined using a NanoDrop 1000 spectrophotometer (Thermo Fisher Scientific, Wilmington, DE) and Bioanalyzer RNA nano chip (Agilent Technologies, Singapore). Only those samples with an RIN between 6 and 7 and a 28S/18 S ratio within the range of 1.5–2 were qualified for cDNA library preparation.

### cDNA library preparation and Illumina sequencing

A total of 5 μg of RNA per sample was used to construct a cDNA library using the NEB Next Ultra RNA Library Prep Kit for Illumina (New England Biol-abs [NEB], USA). Initially, the total RNA sample was digested using DNase I (NEB) and purified using oligo-dT beads (Dynabeads mRNA purification kit, Invitrogen). After mixing with a fragmentation buffer, the mRNA was fragmented into 200–250 bp pieces. Then, cDNA was synthesized using the fragments as templates. Short fragments were purified and resolved with an EB buffer for end reparation and a single nucleotide. Then, short fragments were connected with adapters. Suitable cDNA fragments were selected for PCR amplification. An Agilent 2100 bioanalyzer (Agilent Technologies, Palo Alto, CA) and ABI 7500 real-time PCR machine (Applied Biosystems) were used to determine average molecular lengths. Then, qualified libraries were amplified on cBot to generate a cluster on flow cell (TruSeq PE Cluster Kit V3-cBot-HS, Illumina), and paired-end clusters amplified using flow cell were sequenced on a HiSeq 2000 system (TruSeq SBS KIT-HS V3, Illumina).

### De novo assembly and functional annotation analysis using Illumina sequencing

After sequencing, raw reads were obtained (Accession nos: SRX2676842/SRR5381733 in the NCBI SRA database). First, low-quality, adaptor-polluted, and high-content unknown base (N) reads were filtered to obtain clean reads. De novo assembly of RNA-seq with clean reads was then performed to obtain unigenes using Trinity modules. Sequence splicing and redundancy removing processes were employed. The assembled unigenes was identified the same gene or homolog using a hierarchical clustering approach involving TGICL-CAP3. If the results from any of the databases conflicted with another database result, a priority order was used to decide the sequence direction for the unigenes as follows: NR, Swiss-Prot, KEGG, and COG [34]. Blast2GO [36] was used with NR annotation to obtain GO annotation, and InterProScan5 [37] was used to obtain InterPro annotation.

### Identification of DEGs

To evaluate differences in gene expression during the four developmental periods, an FPKM method (fragments per kilo bases per million reads) was employed to calculate read density. FPKM = 10^9^C/NL, where ‘C’ is the number of mapped fragments for a certain gene, ‘N’ is total reads mapped to the entire genome, and ‘L’ is exon length of a certain gene. A false discovery rate (FDR) was adopted to determine the threshold *P*-value in multiple tests. A stringent cutoff with an FDR of <0.001, P of ≤0.05, and an absolute value of log_2_ ratio of >1 was used to identify differentially expressed genes (DEG). The DEGs were further used for GO and KEGG enrichment analyses.

### Gene coexpression network analysis

According to the result from each sample regarding the unigene expression quantity, we removed the expression quantity from all samples that were <1 and expressions that barely changed (unigene variance was <0.4); the rest of the Unigene as input data was used for gene co-expression network analysis. We computed the correlation for different unigenes using the WGCNA package and obtained a different module. A GO functional analysis and KEGG Pathway analysis were conducted on genes of modules.

### Quantitative real-time*--PCR Validation*

Total RNA was extracted following the previously described methods. Total RNA was reverse transcribed using a ReverTra Ace qPCR RT master mix with a gDNA removal kit (Toyobo, Osaka, Japan). For qRT-PCR, SYBR Green I was added to the reaction mix and performed on a Chromo4 real-time PCR detection system according to the manufacturer’s instructions. The expression level was calculated as 2^−△△Ct^ and normalized to the Ct value of *A. mongolicum* 18S rRNA*.* The qRT-PCR results were obtained from three biological replicates and three technical repeats for each gene and sample.

## Additional files

Additional file 1: Figure S1. Summary of GO terms enriched by total unigenes. (TIFF 6630 kb)

Additional file 2: Figure S2. Summary of pathways enriched by total unigenes. (TIFF 5598 kb)

Additional file 3: Figure S3. Summary of signal transduction pathways for seven hormones. (TIFF 456 kb)

Additional file 4: Figure S4. The list of enriched KEGG pathways. (TIFF 432 kb)

Additional file 5: Figure S5. The summary of enriched KEGG pathways. (TIFF 424 kb)

Additional file 6: Figure S6. GO annotation of Module M20. (TIFF 4404 kb)

Additional file 7: Figure S7. KEGG annotation of Module M20. (TIFF 4300 kb)

## Abbreviations

AFP: Antifreeze proteins
DEGs: Differentially expressed genes
