# Supplementary figures and images for: Genome-wide transcriptome profiling provides overwintering mechanism of Agropyron mongolicum
Source: BMC Plant Biol. 2017 Aug 10;17:138. doi: 10.1186/s12870-017-1086-3 (PMC5553669; doi:10.1186/s12870-017-1086-3)

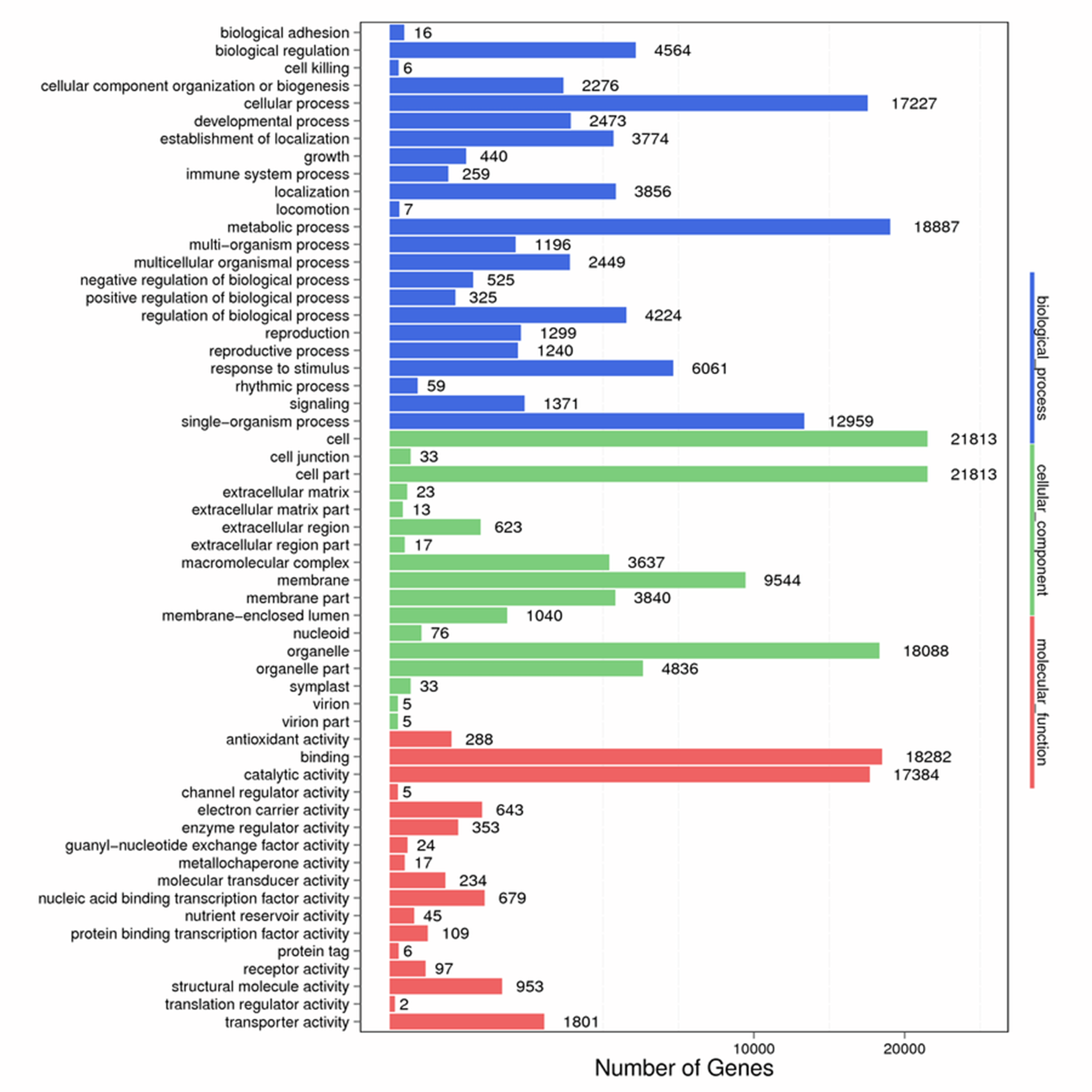

Supplement: Supplementary file 1 — Summary of GO terms enriched by total unigenes. (TIFF 6630 kb) [file 12870_2017_1086_MOESM1_ESM.tif]

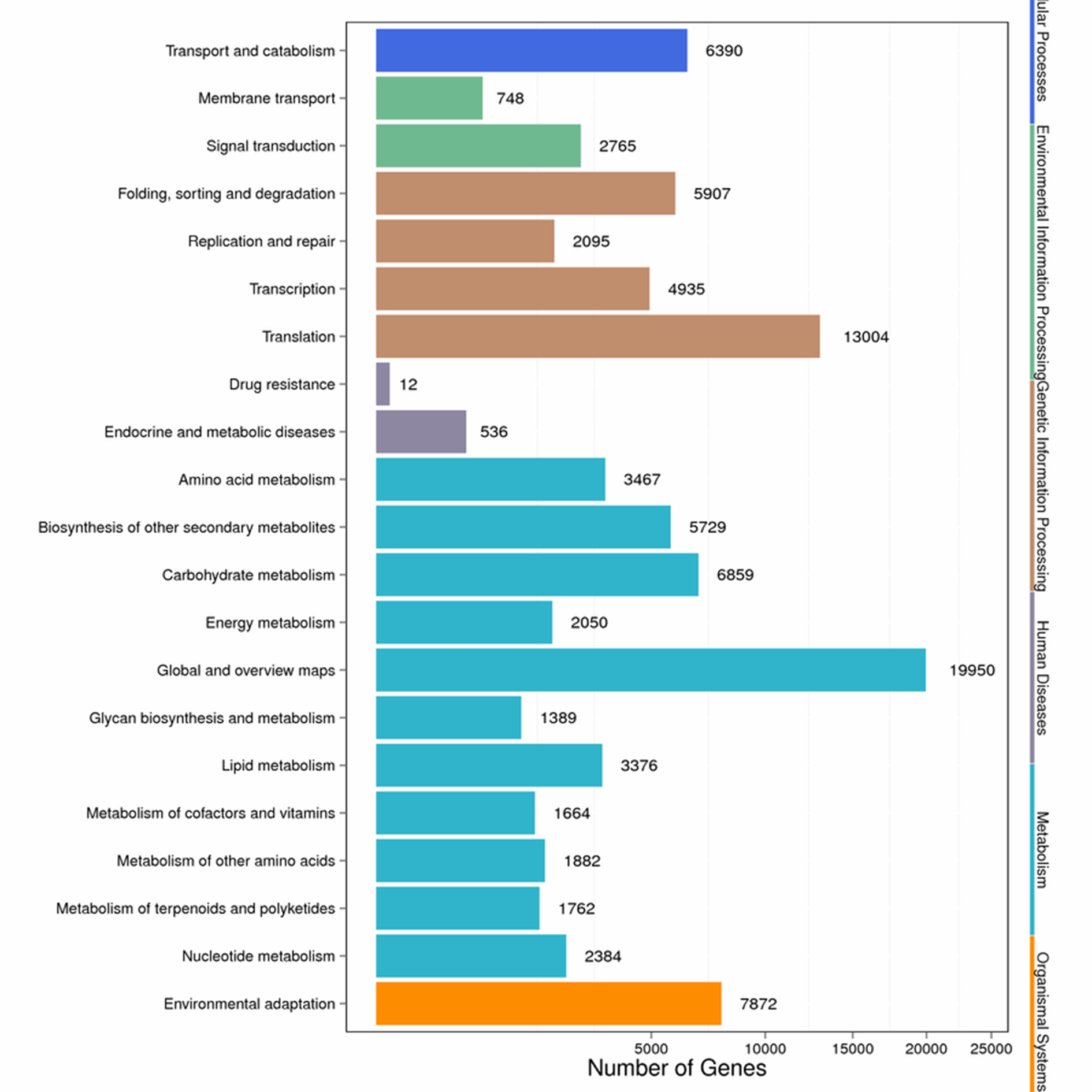

Supplement: Supplementary file 2 — Summary of pathways enriched by total unigenes. (TIFF 5598 kb) [file 12870_2017_1086_MOESM2_ESM.tif]

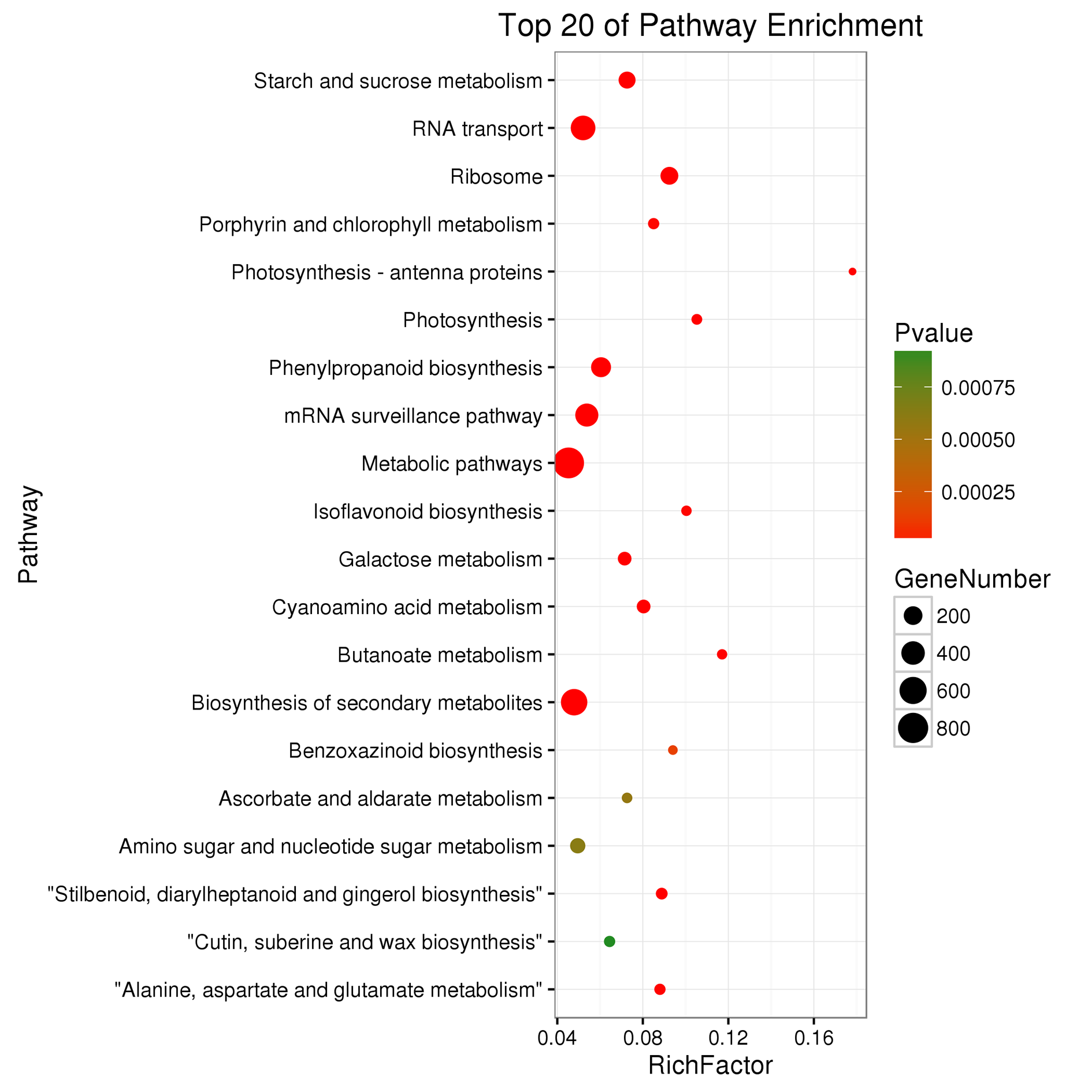

Supplement: Supplementary file 3 — Summary of signal transduction pathways for seven hormones. (TIFF 456 kb) [file 12870_2017_1086_MOESM3_ESM.tif]

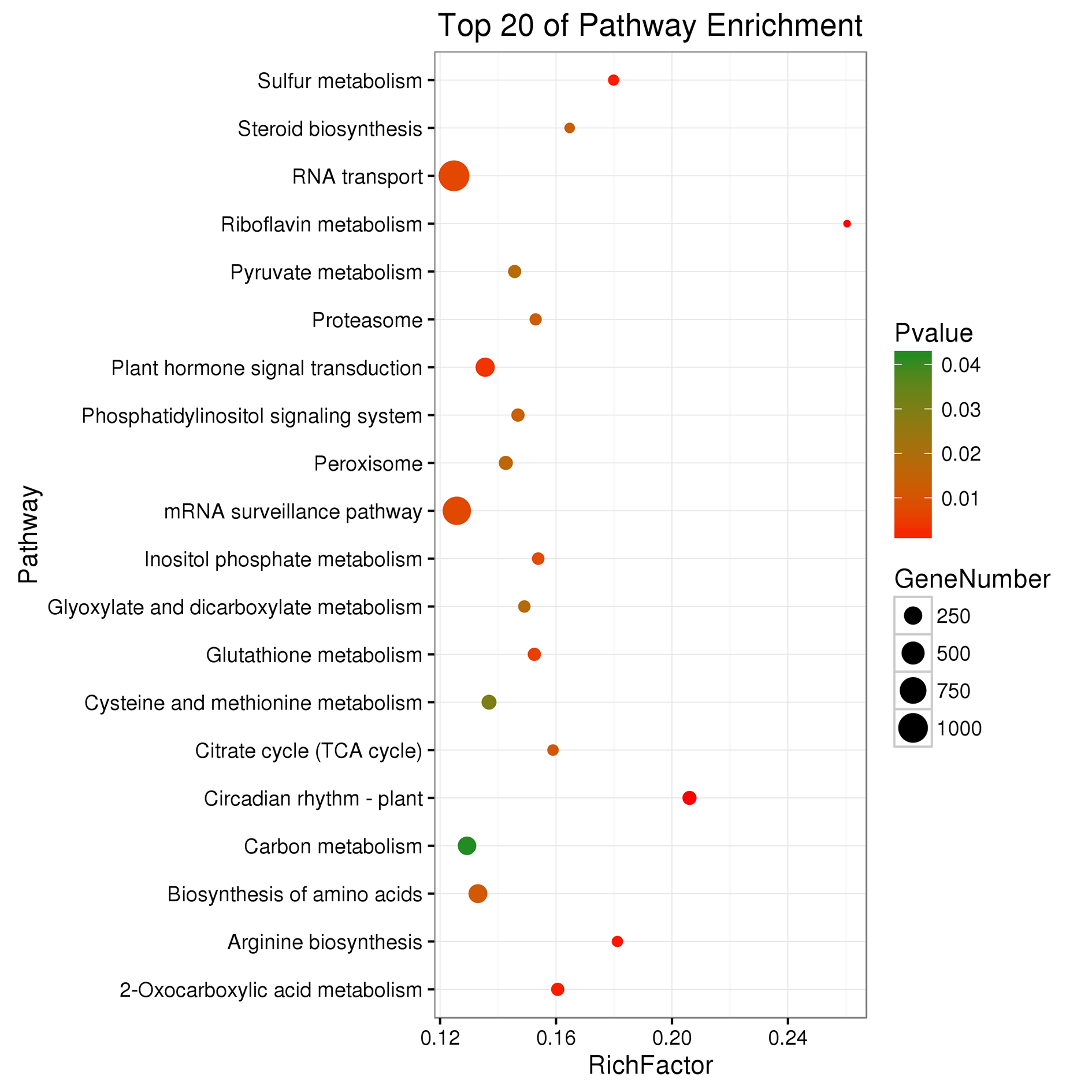

Supplement: Supplementary file 4 — The list of enriched KEGG pathways. (TIFF 432 kb) [file 12870_2017_1086_MOESM4_ESM.tif]

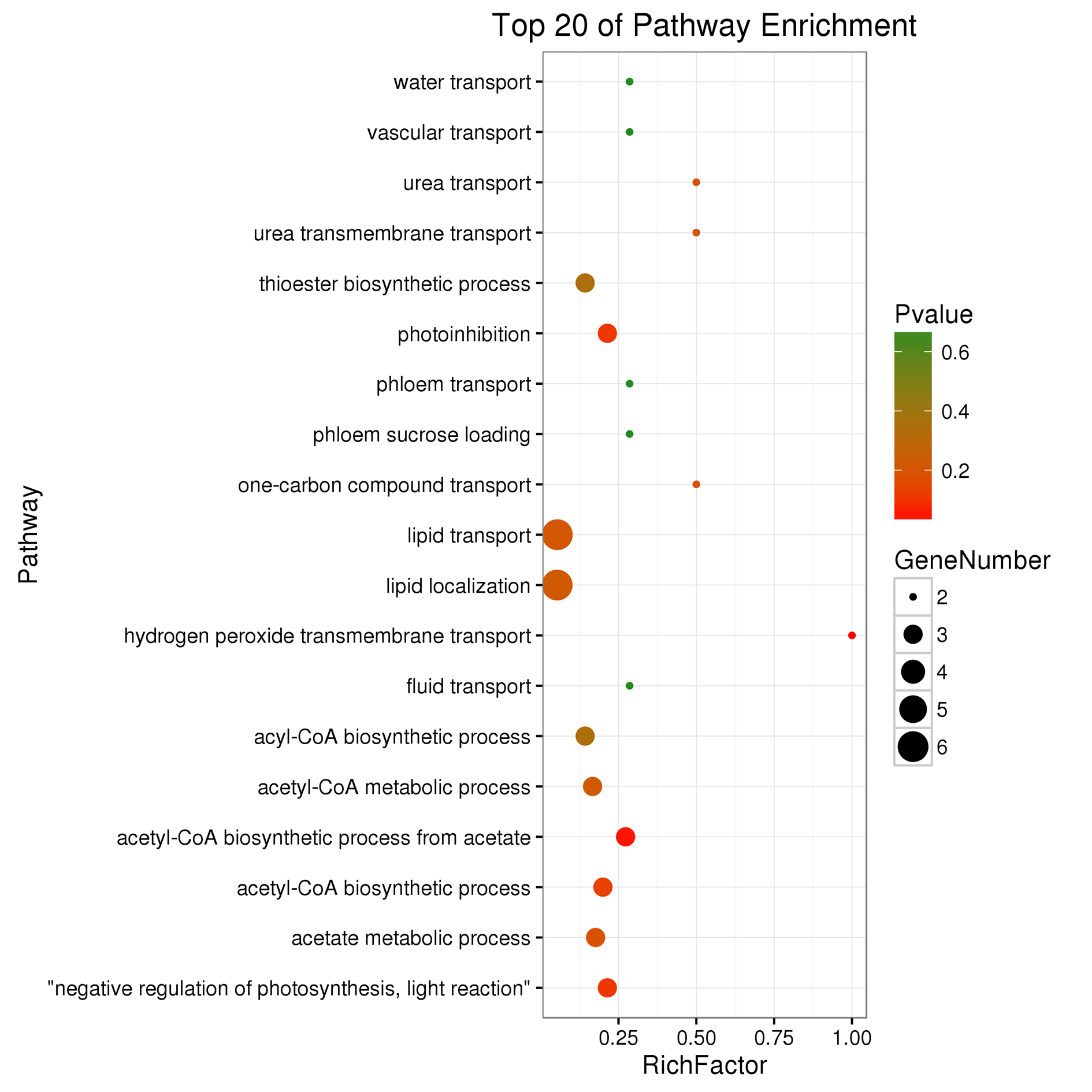

Supplement: Supplementary file 5 — The summary of enriched KEGG pathways. (TIFF 424 kb) [file 12870_2017_1086_MOESM5_ESM.tif]

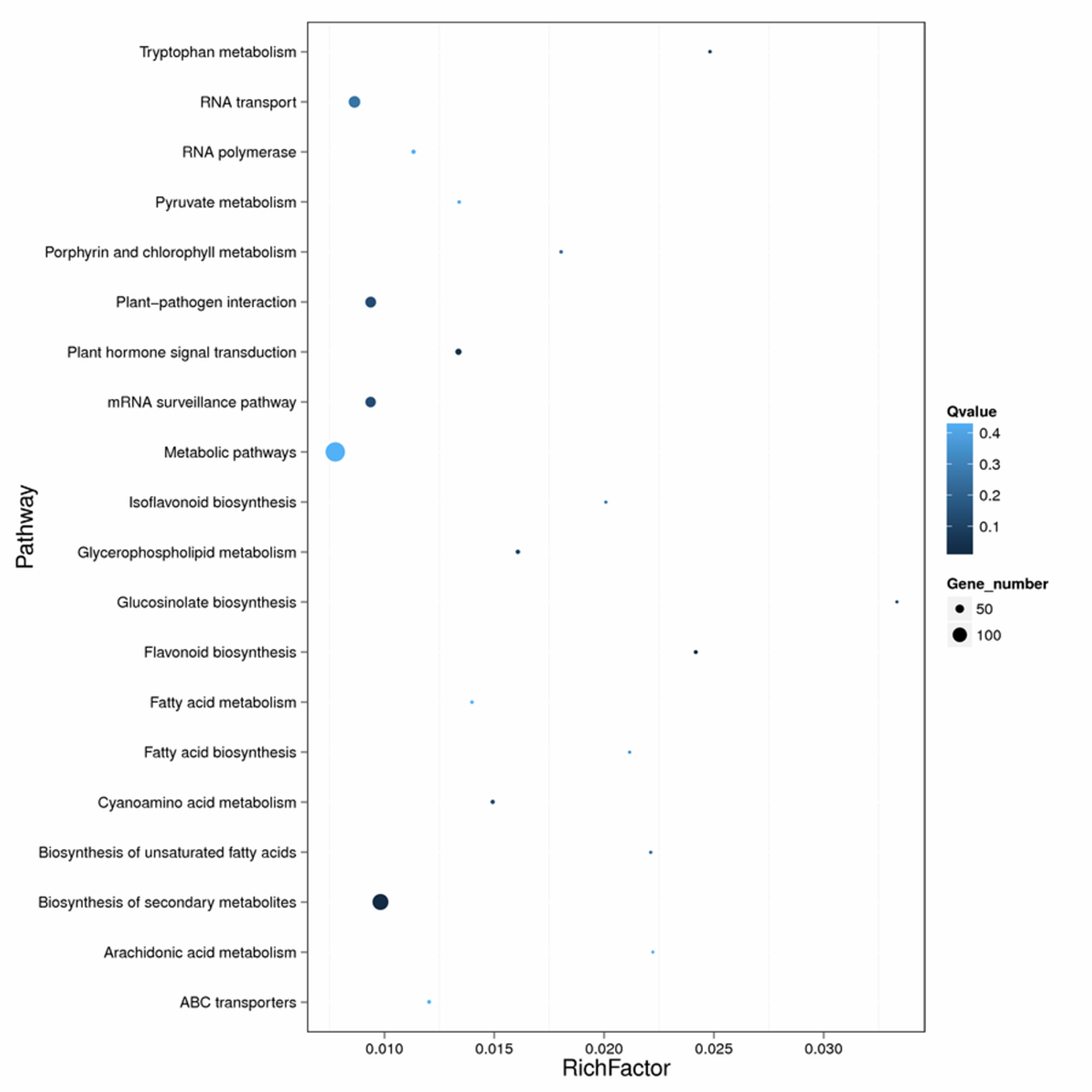

Supplement: Supplementary file 6 — GO annotation of Module M20. (TIFF 4404 kb) [file 12870_2017_1086_MOESM6_ESM.tif]
